# Development and validation of a prediction model to estimate risk of acute pulmonary embolism in deep vein thrombosis patients

**DOI:** 10.1038/s41598-021-04657-y

**Published:** 2022-01-13

**Authors:** You Li, Yuncong He, Yan Meng, Bowen Fu, Shuanglong Xue, Mengyang Kang, Zhenzhen Duan, Yan Chen, Yifan Wang, Hongyan Tian

**Affiliations:** 1 Department of Peripheral Vascular Diseases, The First Affiliated Hospital of Xi’an Jiaotong University, No. 277, Yanta West Road, Xi’an, 710061 Shaanxi China; 2 School of Mathematics, Sun Yat-Sen University, Guangzhou, Guangdong China

**Keywords:** Vascular diseases, Experimental models of disease, Risk factors, Diagnosis, Disease prevention

## Abstract

Venous thromboembolism (VTE), clinically presenting as deep vein thrombosis (DVT) or pulmonary embolism (PE). Not all DVT patients carry the same risk of developing acute pulmonary embolism (APE). To develop and validate a prediction model to estimate risk of APE in DVT patients combined with past medical history, clinical symptoms, physical signs, and the sign of the electrocardiogram. We analyzed data from a retrospective cohort of patients who were diagnosed as symptomatic VTE from 2013 to 2018 (n = 1582). Among them, 122 patients were excluded. All enrolled patients confirmed by pulmonary angiography or computed tomography pulmonary angiography (CTPA) and compression venous ultrasonography. Using the LASSO and logistics regression, we derived a predictive model with 16 candidate variables to predict the risk of APE and completed internal validation. Overall, 52.9% patients had DVT + APE (773 vs 1460), 47.1% patients only had DVT (687 vs 1460). The APE risk prediction model included one pre-existing disease or condition (respiratory failure), one risk factors (infection), three symptoms (dyspnea, hemoptysis and syncope), five signs (skin cold clammy, tachycardia, diminished respiration, pulmonary rales and accentuation/splitting of P_2_), and six ECG indicators (S_I_Q_III_T_III_, right axis deviation, left axis deviation, S_1_S_2_S_3_, T wave inversion and Q/q wave), of which all were positively associated with APE. The ROC curves of the model showed AUC of 0.79 (95% CI, 0.77–0.82) and 0.80 (95% CI, 0.76–0.84) in the training set and testing set. The model showed good predictive accuracy (calibration slope, 0.83 and Brier score, 0.18). Based on a retrospective single-center population study, we developed a novel prediction model to identify patients with different risks for APE in DVT patients, which may be useful for quickly estimating the probability of APE before obtaining definitive test results and speeding up emergency management processes.

## Introduction

Pulmonary embolism (PE) is a common emergency and critical illness in clinical practice, with a sudden dramatic onset and often results in poor outcomes. Clinical evidence shows that about 90% of PE originates from the crumbling away and migration of deep vein thrombosis (DVT), both are collectively referred to as venous thromboembolism (VTE), it represents globally the third most frequent acute cardiovascular syndrome, behind myocardial infarction and stroke^1^. Acute pulmonary embolism (APE) is the most serious clinical type of VTE, which means a common complication of hospitalized patients. Because of occult onset and nonspecific symptoms, APE is usually ignored, which is an important cause of unexpected death and perioperative death of hospitalized patients, and also the main cause of increased medical expenses, extended hospital stay and medical disputes^1–3^. Therefore, how to achieve early identification and diagnosis, timely and effective treatment, standardized follow-up and management to reduce the mortality and recurrence rate of pulmonary embolism patients and improve the prognosis is a major health problem facing China, even the world.

The standard diagnosis of PE mainly includes pulmonary angiography and computed tomography pulmonary angiography (CTPA). They are both very sensitive imaging modality, which has been invaluable tools in the diagnostic work-up and management of patients with suspected PE. However, in the real clinical world, establishing an examination diagnosis for suspected patients is difficult in many low-resource settings, where diagnostic tests are typically unavailable because of a lack of equipment and trained personnel^4^ and prohibitive costs^5^. Other relatively economical and accurate examination means are difficult to be implemented quickly. The results of prospective studies and guidelines lend support to the concept that clinical probability assessment is a fundamental step in the diagnosis of pulmonary embolism^1,6,7^. The pulmonary embolism risk assessment scale recommended by the current guidelines mainly includes the Wells score^8^ and the revised Geneva score^9^. In the Chinese population, the diagnostic value of the Wells scores and revised Geneva score still needs to be verified by multi-center, prospective validation studies in a large cohort. Although some studies demonstrated the usefulness of these traditional scores for identifying suspected patients at risk of developing PE, however, as one of most important inducements of PE, they are not focused on accurately estimating risk in DVT patients.

Based on clinical characteristics and pathogenesis, the primary aim of our study is to estimate the risk of APE in DVT patients. The secondary aim is to develop and validate a predictive model using clinically variables which are readily available in primary care institutions and different professional departments at the time of thrombotic events. To enhance visual presentation and facilitate subsequent clinical applications, heatmap shows the distribution of all the sample's predictor variables, and generated nomograph provides a quick visual technique to assess the clinical probability of acute pulmonary embolism, which can direct personalised decision-making for preventative therapy.

## Methods

### Study design and data source

**Figure 1:**
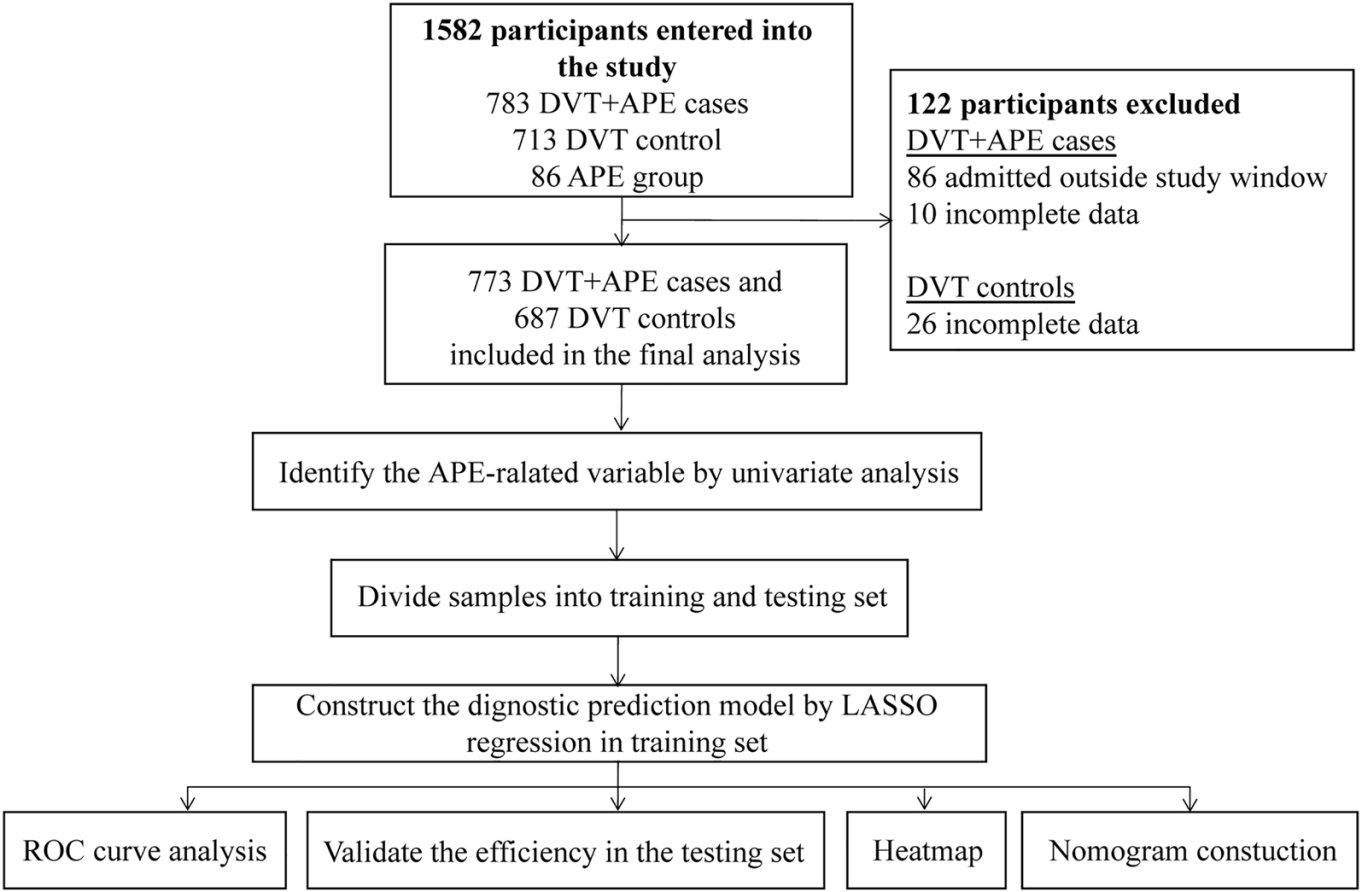
Flow diagram of the overall procedures. DVT, deep vein thrombosis; APE, acute pulmonary embolism; VTE, venous thromboembolism; LASSO, Least absolute shrinkage and selection operator.

Figure 1 illustrates the workflow. The experiments, including any relevant details, were approved by the Ethics Committee of the First Affiliated Hospital of Xi’an Jiaotong University (No: XJTU1AF2018LSK-144) (Supplementary Information 1). The study was performed in accordance with relevant guidelines and regulations. Verbal informed consent was obtained from the patient(s) for their anonymized clinical information to be published in this article. Consecutive patients who were diagnosed as symptomatic VTE between June 1, 2013 and June 1, 2018 (n = 1582) at The First Affiliated Hospital of Xi’an Jiaotong University were initially enrolled in this study. Among them, 122 patients were excluded, including 26 DVT controls (incomplete data in 26), 96 DVT + APE cases (admitted outside study window in 86 and incomplete data in 10). Therefore, a total of 1,460 patients was determined as the required sample size to be enrolled in the study (Supplementary Information 2). Department of Peripheral Vascular Diseases undertakes the important task of diagnosis, treatment and follow-up VTE patients in the whole hospital. So about 30% of patients were suspected and diagnosed of VTE during hospitalization in other departments (such as Department of Critical Care Medicine, Department of Respiratory Medicine, Department of Cardiovascular Medicine, Department of Oncology and so on) or emergency departments of The First Affiliated Hospital of Xi’an Jiaotong University due to other diseases, and then transferred to Department of Peripheral Vascular Diseases for treatment. Approximately 70% of patients presented directly to the Department of Peripheral Vascular Diseases for suspected VTE, which was clearly diagnosed and treated during hospitalization. All enrolled patients confirmed and diagnosed carried out at our institution dedicated diagnostic unit, including pulmonary angiography or CTPA, compression venous ultrasonography and electrocardiogram (ECG). Each patient was examined at baseline according to a standardized protocol, following recommended international standards. Pulmonary angiography was performed after obtaining written informed consent from the patients. Exclusion criteria were: (1) recurrent pulmonary embolism, (2) incomplete clinical data, (3) contraindication to CTPA/pulmonary angiography, (4) the patient refuses to complete diagnostic test.

### Predictor variables

In the first step, we searched PubMed and Web of Science databases without language or time restrictions to retrieve relevant studies. The prediction factors were mainly derived from 2019 ESC Guidelines for the diagnosis and management of acute pulmonary embolism^1^ and a systematic review and meta-analysis that was designed to identify factors for VTE in hospitalized medical patients^10^. To maximize safety and model usability, we tend to choose reasonable and clinically relevant predictors that are easily available in primary care institutions and different professional departments. Because some biochemical tests are not routinely available, we did not consider biomarkers endorsed by guidelines (D-dimer or pro-BNP). Age as one of continuous variables, was transformed into binary variables using pre-specified cut-offs either (> 65 years and < 65 years) derived from literature^9^. Meta-analysis found low-certainty evidence of association between risk of any VTE and central venous catheters (CVC) use^10^, we did not choose CVC use, this risk factor is less common in our sample population. There is probably an association between risk of any VTE and elevated heart rate(> 100 beats per minute), therefore, we selected tachycardia (> 100 beats per minute) and heart rate (as continuous variables).

Based on literature and research reports, we screened more than 10 kinds of electrocardiogram sign associated with APE^11–15^. The ECGs obtained within the first 24 h of hospital admission were included in the study. Patients with acute cor pulmonale were deemed present if we identified at least one of the following: (1) S_I_Q_III_T_III_, (2) T-wave inversion in right precordial leads, (3) S_1_S_2_S_3_, (4) pseudo infarction, (5) transient right bundle branch block. If the above signs had appeared in the past, they would be excluded.

In order to control bias and produce reliable data for research, the following rules were decided upon: (1) The principle of blind method was guaranteed in the whole process of experimental design, study implementation and statistical analysis. (2) At the time of diagnosis, all the eligible cases undergone by trained clinical doctors to determine the presence or absence of signs and symptoms related to VTE, as dichotomous variables (yes/no), including dyspnea, hemoptysis, chest pain, syncope, swelling pain in the lower limbs and so on. (3) The clinical doctor should be careful to identify potential factors associated with APE and exclude pre-existing medical history, that are similar to the clinical manifestations of pulmonary embolism. (4) Trained research personnel completed a training course designed to explain all variables to ensure that the same data collection methods were followed. (5) Clinical data and inspection results were abstracted from hospital medical records by trained research personnel using a standardized form and they did not be aware of the final diagnosis at the time of data collection. (6) The data collector was not allowed to take charge of data analysis and did not know the research protocol. (7) The data collector ensured fully understood all eligible patients' clinical data and the survey was conducted in a quiet room without any disturbance. (8) The statistician analyzed the data independently without any disturbance.

### Outcome variables

The primary outcomes of this study were as follows: (1) an easy-to-use predictive model for acute pulmonary embolism was derived and validated, (2) a reasonable pipeline of disease risk prediction and factor analysis was introduced.

### Derivation and validation of the models

The initial cohort comprised 1582 symptomatic VTE patients. 36 patients (including 10 DVT + APE cases and 26 DVT controls) were excluded due to incomplete data, 86 were excluded due to acute pulmonary embolism only, therefore, 1460 patients (DVT + APE vs DVT 773:687) were included in this study. Then, we randomly classified samples as training set (1095) and testing set (365) in a 3:1 ratio. The training set was used to generate the prediction model, and testing set was used to evaluate the prediction performance of the model. Firstly, we performed univariate analysis to select predictor variables those significantly linked with APE diagnosis, using a cutoff of p < 0.05 (Supplementary Tables 1 and 2). To avoid overfitting, LASSO regression analysis was used to screened those APE diagnostic-related variables. Later, all APE diagnostic-related predictor variables were included in the multivariate analysis to assess independent predictor factors using logistics regression (Supplementary Table 3). Ultimately, we constructed sixteen APE diagnostic-related predictors as candidates for the prediction model. The area under the receiver operator characteristics (AUC) curves was used to evaluate the diagnostic efficiency of the model. Based on the AUC, Brier score and calibration curves were used to evaluate the concordance between predicted diagnosis outcomes in training set and testing set. The prediction model distribution of patients at different risk levels, the number of censored patients, and the heatmap of APE diagnostic-related predictors were displayed. Establishment of the nomogram based on independent risk factors resulting from multivariate logistics regression to predict the APE probabilities for patients with DVT.

All figures were created using R software version 4.0.2. LASSO logistic regression was performed by package 'glmnet' function of 'glmnet' package. The AUC and Brier score for the model were calculated using the R package of the “riskRegression”. The nomogram was constructed using the logistic regression analysis with the R package “rms”.

### Handling of missing data

Except for age and gender, there was tiny missing data for all variables. We eliminated the missing variables and analyzed the complete data.

### Statistical analysis

The statistical analysis was performed in R software (version 4.0.2; https://www.R-project.org). p < 0.05 was considered statistically significant.

## Results

### Study population

Baseline characteristics according to risk groups are shown in Table 1. Among the analyzed patients, 703 patients (48.1%) were males, 757 patients (51.9%) were females. The median age for 687 patients with DVT only was 59 years (interquartile range [IQR], 48–68), and the median age among 773 patients with DVT and PE was 62 (interquartile range [IQR], 51–70). The overall prevalence of pulmonary embolism was 52.9% (773 of 1460 patients).

**Table 1:** Demographic and clinical characteristics of the study patients.

Characteristic n (%) or median (IQR)	DVT	DVT + APE
	n = 687	n = 773
**Sex**		
Male	330 (48)	373 (48)
Female	357 (52)	400 (52)
Age	59 (48, 68)	62 (51, 70)
**Pre-existing disease or condition**		
Heart failure	21 (3.1)	19 (2.5)
Respiratory failure	1 (0.1)	31 (4.0)
Previous history of VTE	76 (11)	43 (5.6)
Autoimmune disease	17 (2.5)	26 (3.4)
Malignant tumor	77 (11)	59 (7.6)
**Risk factors**		
Fracture of lower limb	65 (9.5)	96 (12)
Severe trauma	32 (4.7)	36 (4.7)
Spinal cord injury	5 (0.7)	15 (1.9)
Arthroscopic operation	23 (3.3)	14 (1.8)
Blood transfusion	25 (3.6)	32 (4.1)
Hormone replacement therapy	22 (3.2)	19 (2.5)
Infection	27 (3.9)	96 (12)
Paralytic stroke	36 (5.2)	46 (6.0)
Superficial venous thrombosis	19 (2.8)	6 (0.8)
Postpartum period	21 (3.1)	14 (1.8)
Stay in bed (> 3 days)/undergo surgery	181 (26)	210 (27)
Long time of sitting (> 6 h)	81 (12)	29 (3.8)
Undergo hysteroscopy/Laparoscopy surgery	29 (4.2)	31 (4.0)
Laricose vein of lower limb	46 (6.7)	43 (5.6)
Smoke	192 (28)	195 (25)
**Symptoms**		
Dyspnea	28 (4.1)	302 (39)
Hemoptysis	3 (0.4)	33 (4.3)
Chest pain	13 (1.9)	90 (12)
Swelling and pain in the lower limbs	657 (96)	593 (77)
Fever	32 (4.7)	52 (6.7)
Syncope	8 (1.2)	99 (13)
Cough	33 (4.8)	74 (9.6)
Palpitation	5 (0.7)	31 (4.0)
Delirium/disturbance of consciousness	1 (0.1)	5 (0.6)
**Signs**		
Skin cold clammy	7 (1.0)	27 (3.5)
Cyanosis of the lips	1 (0.1)	19 (2.5)
Tachycardia	36 (5.2)	109 (14)
Diminished respiration	1 (0.1)	43 (5.6)
Pulmonary rales	7 (1.0)	78 (10)
Accentuation/splitting of P_2_	100 (15)	178 (23)
Distention of jugular vein/hepatojugular reflex	2 (0.3)	8 (1.0)
**ECG**		
Heart rate	78 (69, 89)	82 (72, 94)
S_I_Q_III_T_III_	21 (3.1)	137 (18)
Nodal tachycardia	54 (7.9)	103 (13)
Right ventricular hypertrophy	0 (0)	13 (1.7)
Right axis deviation	4 (0.6)	19 (2.5)
Left axis deviation	55 (8.0)	176 (23)
S_1_S_2_S_3_	2 (0.3)	41 (5.3)
Low voltage	17 (2.5)	35 (4.5)
Clockwise rotation of cardiac electric axis	1 (0.1)	9 (1.2)
ST-segment elevation	10 (1.5)	14 (1.8)
ST-segment depression	33 (4.8)	91 (12)
T wave inversion(V_1_–V_3_/V_4_)	34 (4.9)	175 (23)
ST-segment depression(II/III/aVF)	14 (2.0)	71 (9.2)
Q/q wave(II/aVF)	17 (2.5)	74 (9.6)
T wave inversion(II/aVF)	6 (0.9)	56 (7.2)
Right bundle branch block	25 (3.6)	50 (6.5)

*ECG* electrocardiogram, *APE* acute pulmonary embolism, *DVT* deep vein thrombosis, *VTE* venous thromboembolism, *P2* pulmonary valve second heart sound.

### Model development

**Figure 2:**
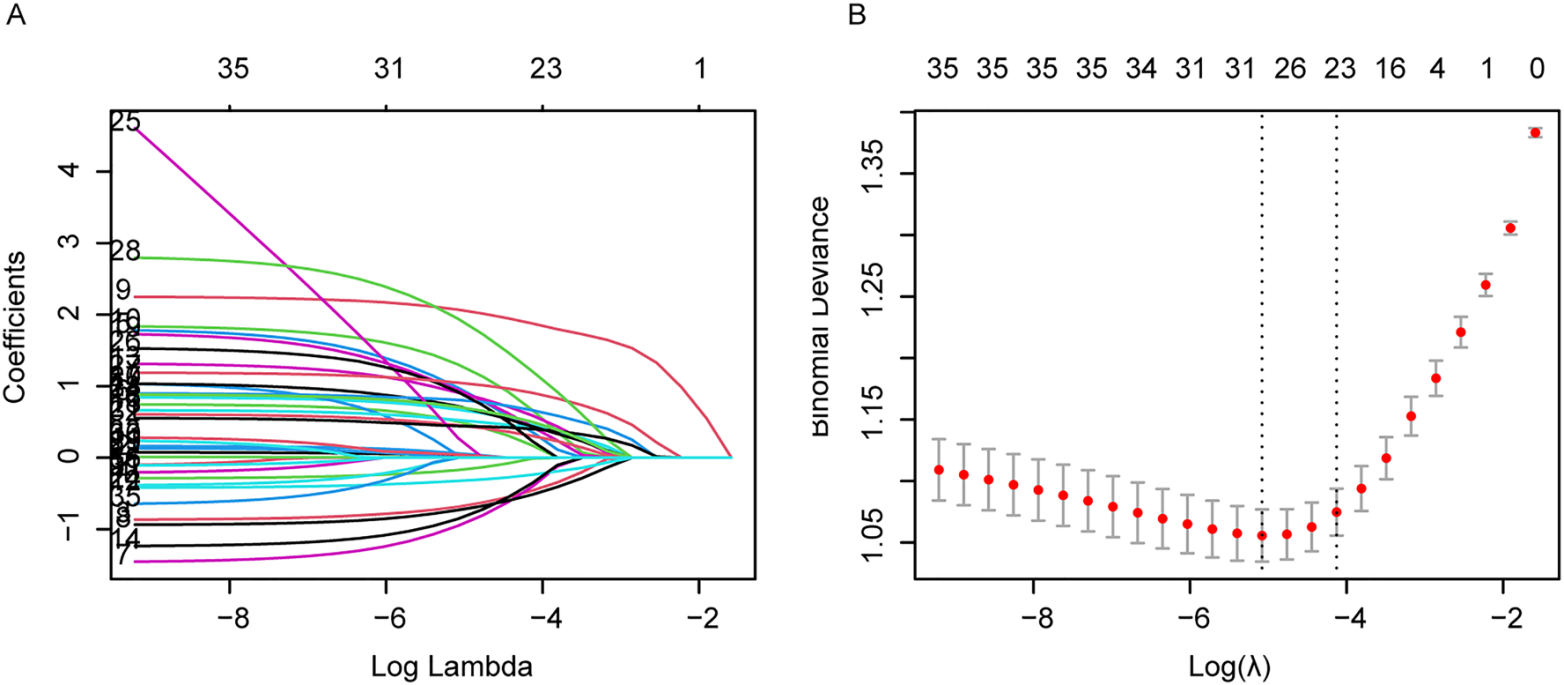
Predictor variables selection using the least absolute shrinkage and selection operator (LASSO) binary logistic regression model. **(A)** LASSO coefficient profiles of the 54 predictor variables. A coefficient profile plot was produced against the log (λ) sequence. Vertical line was drawn at the value selected using tenfold cross-validation, where optimal l resulted in 23 nonzero coefficients. **(B)** Tuning parameter (λ) selection in the LASSO model used tenfold cross-validation via minimum criteria. The area under the receiver operating characteristic (AUC) curve was plotted versus log(λ). Dotted vertical lines were drawn at the optimal values by using the minimum criteria and the 1 standard error of the minimum criteria (the 1-SE criteria). A λ value of 0.011732, with log (λ), −6.413407 was chosen (1-SE criteria) according to tenfold cross-validation. The figures were created using R software v4.0.2.

A total of 54 variables were obtained from systematic review and meta-analysis, which has previously been reported to be associated with VTE. Univariate regression analysis was performed on 54 selected variables. We found that 34 variables were significantly linked with diagnosis of APE in DVT patients (p < 0.05) (Supplementary Tables 1 and 2). Lasso regression analysis and multivariate logistics regression analysis were adopted for the 34 APE diagnostic-related variables (Supplementary Table 3). Based on the results of the univariate analysis, 23 variables are included in the Lasso regression model (Fig. 2). After selecting the above 23 variables through multiple logistic regression again, 20 variables were independently associated with APE. We included 16 variables with OR value > 1 to build a prediction model, and named the model as APE risk prediction model (Table 2).

**Table 2:** The APE risk prediction model based on independent predictors of acute pulmonary embolism in training set.

Characteristic	Coefficient	S.E	OR	95% CI for OR		*p*-value
				Lower	Upper	
**Pre-existing disease or condition**						
Respiratory failure	1.85	1.09	6.38	1.11	122.08	0.09
**Risk factors**						
Infection	0.85	0.32	2.33	1.25	4.42	0.01
**Symptoms**						
Dyspnea	2.14	0.26	8.52	5.22	14.50	< 0.00
Hemoptysis	1.36	0.68	3.89	1.16	17.90	0.05
Syncope	1.38	0.49	3.99	1.63	11.30	0.00
**Signs**						
Skin cold clammy	0.69	0.55	2.00	0.71	6.19	0.20
Tachycardia	0.70	0.27	2.01	1.19	3.44	0.01
Diminished respiration	1.87	1.09	6.49	1.11	124.78	0.09
Pulmonary rales	0.93	0.59	2.53	0.87	9.29	0.12
Accentuation/splitting of P_2_	0.48	0.19	1.62	1.12	2.34	0.01
**ECG**						
S_I_Q_III_T_III_	1.00	0.32	2.71	1.46	5.23	0.00
Right axis deviation	1.52	0.86	4.58	0.96	33.04	0.08
Left axis deviation	1.14	0.22	3.11	2.03	4.84	0.00
S_1_S_2_S_3_	2.78	1.06	16.16	3.04	299.29	0.01
T wave inversion(V_1_–V_3_/V_4_)	0.64	0.27	1.89	1.12	3.24	0.02
Q/q wave(II/aVF)	0.91	0.40	2.49	1.16	5.63	0.02
Constant	−1.00	0.10	0.37	0.30	0.45	< 0.00

*CI* confidence interval.

**Figure 3:**
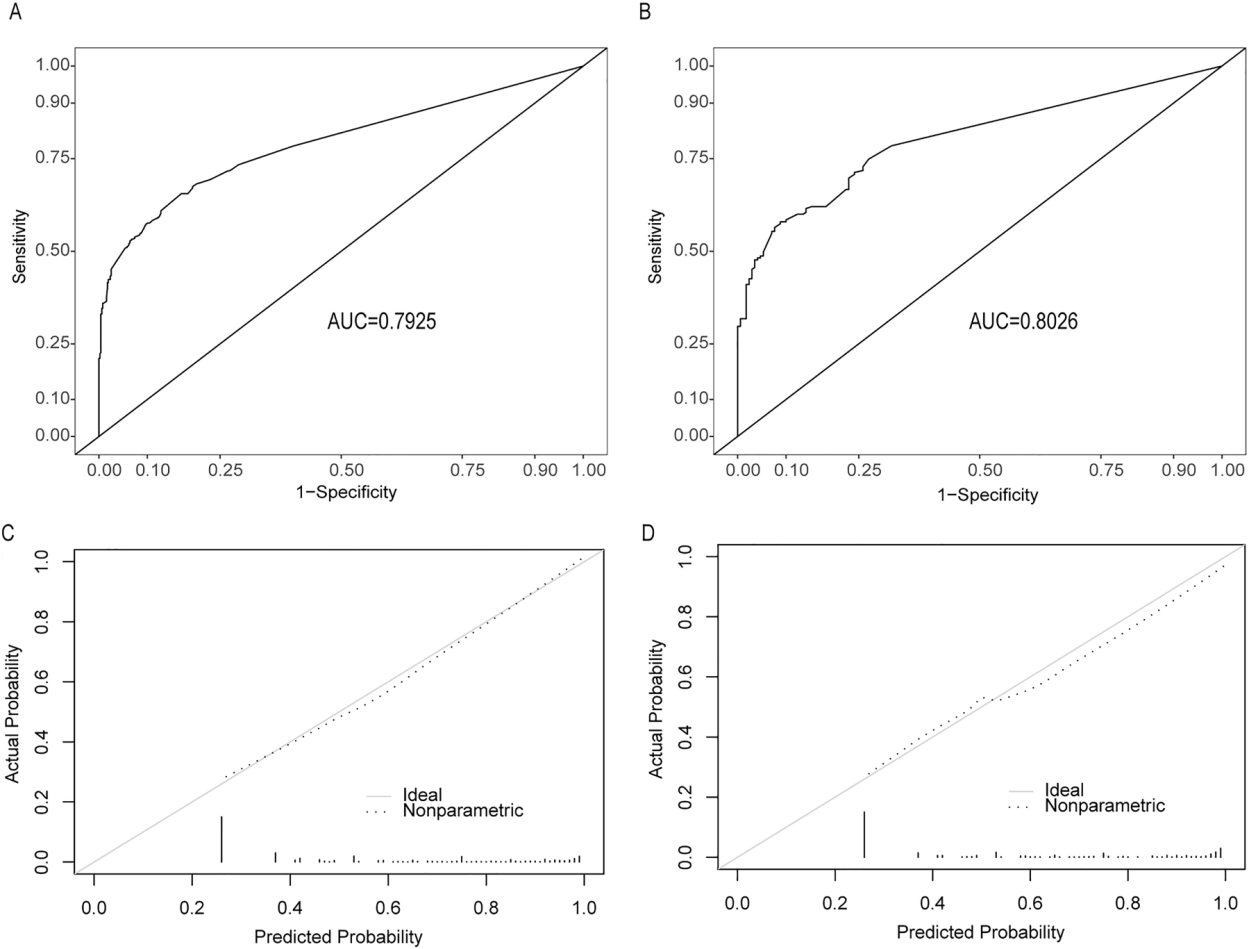
ROC curves and calibration curve of the APE risk prediction model. **(A)** ROC curve and corresponding AUC for the prediction model of APE diagnosis in the training set. **(B)** ROC curve and corresponding AUC for the prediction model of APE diagnosis in the testing set. **(C)** The calibration curve of training set. **(D)** The calibration curve of testing set. *ROC* receiver operator characteristics, *AUC* area under the receiver operator characteristics curves. The figures were created using R software v4.0.2.

The APE risk prediction model included one pre-existing disease or condition(respiratory failure),one risk factors(infection), three symptoms(dyspnea, hemoptysis and syncope), five signs(skin cold clammy, tachycardia, diminished respiration, pulmonary rales and accentuation/splitting of P_2_), and six ECG indicators(S_I_Q_III_T_III_, right axis deviation, left axis deviation, S_1_S_2_S_3_, T wave inversion and Q/q wave), of which all were positively associated with APE in DVT patients. The area under the ROC curve was 0.79 (95% CI, 0.77–0.82) (Fig. 3).

### Internal validation

To validate the APE risk prediction model, we used an internal validation procedure based on random classify validation. The ROC curves of the model showed AUC of 0.79 (95% CI, 0.77–0.82) and 0.80 (95% CI, 0.76–0.84) in the training set and testing set, respectively, and no significant difference was found between these values, indicating the reliability of the nomogram (Fig. 3). This model had a Brier score of 0.18, calibration slope of 0.83, indicating good predictive accuracy performance (Fig. 3).

### Model presentation

**Figure 4:**
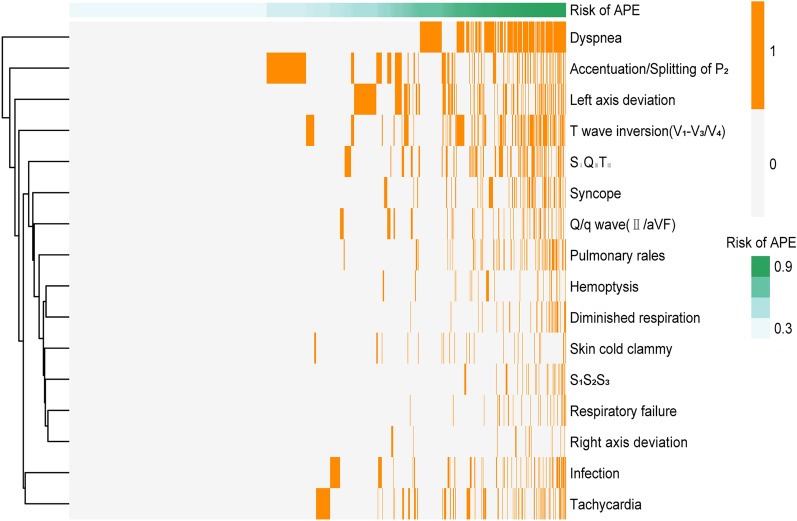
Heatmap to display the occurrence of the individual predictor variables for each sample. The figures were created using R software v4.0.2.

**Figure 5:**
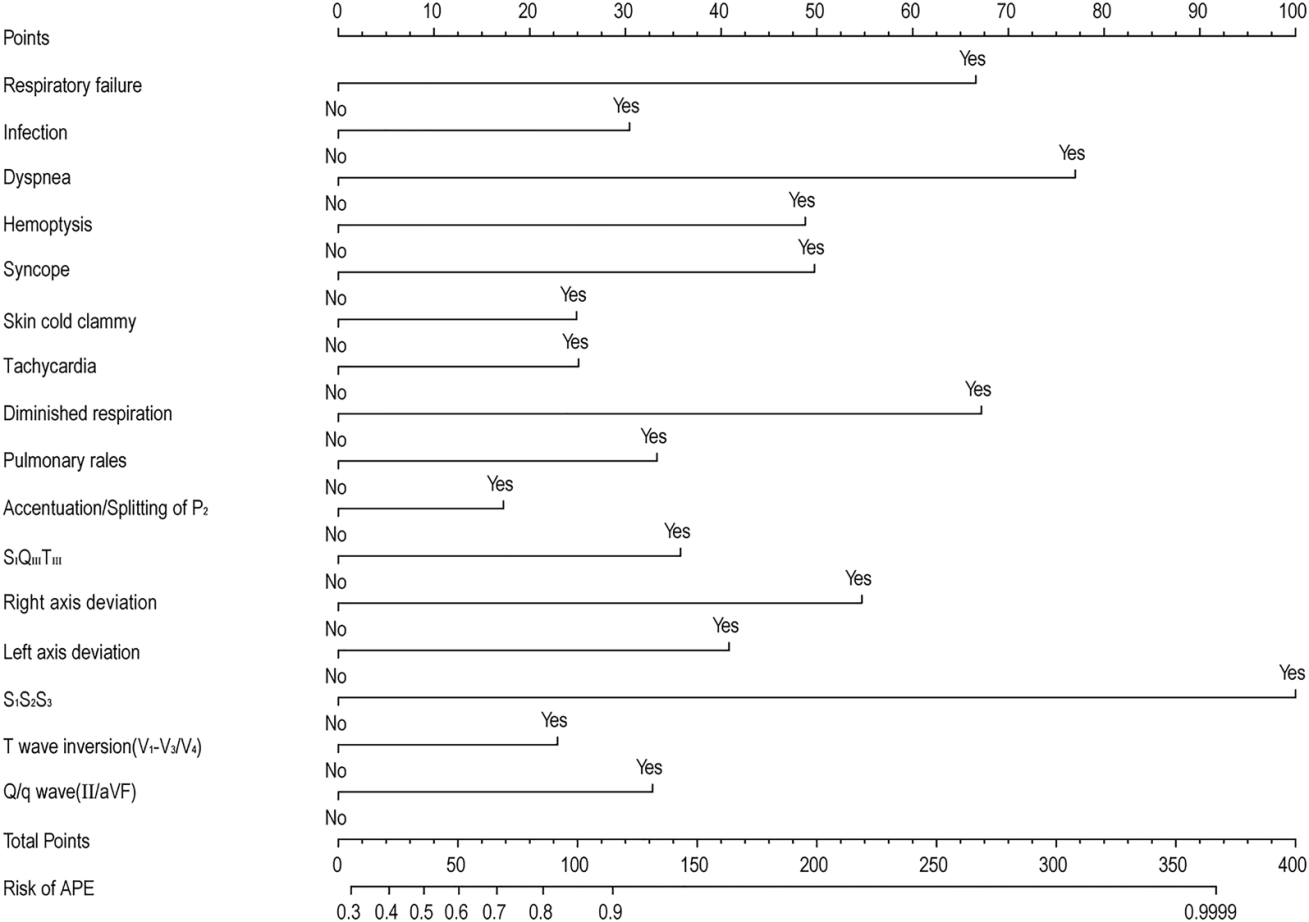
Nomogram to estimate the probability of acute pulmonary embolism. The figures were created using R software v4.0.2.

Since none of the prediction models performed well in all patients with APE, we try to derive a new predictive model which better identify patients at risk of deterioration. Our model had a good discriminatory power for APE in DVT patients (AUC, 0.79; 95% CI, 0.77–0.82). Heatmap showed that high-risk patients had more kinds of risk factors, which suggested that there were significant differences between the 16 diagnostic-related variables in high-risk and low-risk score APE patients (Fig. 4). To generate and validate an APE risk prediction model that could be translated to the clinic, we developed a nomogram to predict risk of APE in DVT patients (Fig. 5).

## Discussion

Utilizing high-quality data from a retrospective cohort study, we derived an easy-to-use clinical score to predict the risk of developing APE in the DVT patients. The APE risk prediction model derived from a large cohort of consecutive inpatient with diagnostic examination, totally based on past medical history, clinical symptoms, physical signs, and the sign of the electrocardiogram. Sixteen clinical predictors accurately identified patients with high-risk disease who may benefit from individualized management to improve clinical outcomes. The excellent discriminatory power of our model was validated by internal validation.

We purposefully selected to utilize readily available predictors to enhance clinical applicability and ease of use, particularly for primary care institutions and different professional departments. In our study, sudden-onset dyspnea and hemoptysis are powerful predictors of pulmonary embolism, it is consistent with previous reports^16,17^. Pulmonary embolism was identified in nearly one of every six patients hospitalized for a first episode of syncope^18^, therefore, syncope was selected as a predictive variable and was eventually included in the predictive model. Right ventricular dysfunction is associated with thrombotic load and one of the important prognostic factors of pulmonary embolism. In APE patients, there is always at least one ECG sign of right ventricular strain, including S_I_Q_III_T_III_, right bundle branch block and T wave inversions^14^. Our model included a total of 6 ECG sign, and these indicators have previously been reported to be related to pulmonary embolism.

Why choose Electrocardiogram as a predictor of APE? Electrocardiogram is an irreplaceable examination method to explore and measure abnormal electrocardiogram activity. It has the advantages of non-invasive, timeliness and simple operation, and has become a necessary examination for patients with unexplained dyspnea or chest pain^19^. The changes of electrocardiogram frequency, rhythm and conduction in APE patients, throughout the disease course and during treatment phases, may better assess risk stratification, prognosis and outcome of the disease and hence the opportunity for more applicable and balanced targeted preventative strategies^13,20,21^.

Is this prediction model clinically generalized? Firstly, as one of the most common clinical examination methods, electrocardiogram is often used to assist early screening of suspected patients. Typically, the sign of the electrocardiogram requires physicians to provide scientific and medical expertise, and the electrocardiographic abnormalities with acute cor pulmonale are well-defined criteria, which have been known and applied for many years^22^. Except electrocardiogram, all the data required for the prediction model are routinely collected in the context of suspected acute pulmonary embolism and are available from the patient’s history and physical examination. Since the model was derived from multidisciplinary patients, we believe that the prediction model is applicable to all clinical departments and easy to calculate.

Is this prediction model valid and accurate? In terms of prediction accuracy, all patients received a diagnosis by a gold standard criterion, and our prediction model could be considered accurate for predicting pulmonary embolism and superior to other people's reports. The model displayed good discrimination in the training set and testing sets (area under ROC curve, 0.79 and 0.80, respectively).

In fact, our prediction model is being extended and externally validated in multiple centers. Preliminary experimental results prove the feasibility of our ideas, the ability of the model to distinguish patients’ risk for APE in the validation cohort is at least as good as in the original cohort. To facilitate clinical visualization management, instead of using points proportional to their beta regression coefficient values, we estimate the probability of acute pulmonary embolism directly from Nomogram.

There are potential limitations to our study. Firstly, the study is a retrospective study and not a population-based study or nationwide survey, which had an unavoidable selection bias. Secondly, the original intention of this model was to serve primary care institutions and simplify the diagnosis process, so we did not include the biochemical indicators recommended by the guidelines, such as D-dimer, pro-BNP, etc. Finally, as is often the case in clinical diagnostic studies, in our study, we did not account for the uncertainty around predictions, but focused on the clinical possibility assessment. Hopefully, this model will be further validated in a large, multi-center, prospective validation study before providing benefits for Chinese patients.

In conclusion, this study reports the derivation and initial validation of a sixteen variable clinical prediction model that demonstrated good overall accuracy in predicting risk of acute pulmonary embolism for patients with deep vein thrombosis. The above means the prediction model appears more suitable for primary care institutions and different professional departments. Pending external validation, this study now provides the basis and information for risk assessment of patients with acute pulmonary embolism.

## Supplementary Information

Supplementary Table 1.

Supplementary Table 2.

Supplementary Table 3.

Supplementary Information 1.

Supplementary Information 2.

## Data Availability

All data generated or analyzed during this study are included in this published article.
