# Supplementary material for: Development and validation of a prediction model to estimate risk of acute pulmonary embolism in deep vein thrombosis patients
Source: Sci Rep. 2022 Jan 13;12:649. doi: 10.1038/s41598-021-04657-y (PMC8758720; doi:10.1038/s41598-021-04657-y)
Supplement: Supplementary file 1 — Supplementary Table 1. [file 41598_2021_4657_MOESM1_ESM.docx]

Appendix Table 1 Univariate analysis of clinical predictors

| **Characteristic**  n (%) or median (IQR) | **DVT (n=687)** | | | **DVT+PE (n=773)** | | ***p-Value*** |
| --- | --- | --- | --- | --- | --- | --- |
|  | No | Yes | No | | Yes |  |
| Sex |  |  |  | |  |  |
| Male | 330 (48.0) | | | 373 (48.3) | | 0.975 |
| Female | 357 (52.0) | | | 400 (51.7) | |  |
| Age | 59.0(48.0, 68.0) | | | 62.0 (51.0, 70.0) | | 0.001 |
| **Pre-existing disease or condition** |  |  |  | |  |  |
| Heart failure | 666 (96.9) | 21 (3.1) | 754 (97.5) | | 19 (2.5) | 0.59 |
| Respiratory failure | 686 (99.9) | 1 (0.1) | 742 (96.0) | | 31 (4.0) | <0.001 |
| Previous history of VTE | 611 (88.9) | 76 (11.1) | 730 (94.4) | | 43 (5.6) | <0.001 |
| Autoimmune disease | 670 (97.5) | 17 (2.5) | 747 (96.6) | | 26 (3.4) | 0.397 |
| Malignant tumor | 655 (95.3) | 32 (4.7) | 737 (95.3) | | 36 (4.7) | 1 |
| **Risk factors** |  |  |  | |  |  |
| Fracture of lower limb | 622 (90.5) | 65 (9.5) | 677 (87.6) | | 96 (12.4) | 0.086 |
| Severe trauma | 655 (95.3) | 32 (4.7) | 737 (95.3) | | 36 (4.7) | 1 |
| Spinal cord injury | 682 (99.3) | 5 (0.7) | 758 (98.1) | | 15 (1.9) | 0.078 |
| Arthroscopic operation | 664 (96.7) | 23 (3.3) | 759 (98.2) | | 14 (1.8) | 0.089 |
| Blood transfusion | 662 (96.4) | 25 (3.6) | 741 (95.9) | | 32 (4.1) | 0.721 |
| Hormone replacement therapy | 665 (96.8) | 22 (3.2) | 754 (97.5) | | 19 (2.5) | 0.484 |
| Infection | 660 (96.1) | 27 (3.9) | 677 (87.6) | | 96 (12.4) | <0.001 |
| Paralytic stroke | 651 (94.8) | 36 (5.2) | 727 (94.0) | | 46 (6.0) | 0.635 |
| Superficial venous thrombosis | 668 (97.2) | 19 (2.8) | 767 (99.2) | | 6 (0.8) | 0.006 |
| Postpartum period | 666 (96.9) | 21 (3.1) | 759 (98.2) | | 14 (1.8) | 0.167 |
| Stay in bed(>3 days)/Undergo surgery | 506 (73.7) | 181 (26.3) | 563 (72.8) | | 210 (27.2) | 0.769 |
| Long time of sitting(>6 hours) | 606 (88.2) | 81 (11.8) | 744 (96.2) | | 29 (3.8) | <0.001 |
| Undergo hysteroscopy/Laparoscopy surgery | 658 (95.8) | 29 (4.2) | 742 (96.0) | | 31 (4.0) | 0.944 |
| Laricose vein of lower limb | 641 (93.3) | 46 (6.7) | 730 (94.4) | | 43 (5.6) | 0.427 |
| Smoke | 495 (72.1) | 192 (27.9) | 578 (74.8) | | 195 (25.2) | 0.264 |
| **Symptoms** |  |  |  | |  |  |
| Dyspnea | 659 (95.9) | 28 (4.1) | 471 (60.9) | | 302 (39.1) | <0.001 |
| Hemoptysis | 684 (99.6) | 3 (0.4) | 740 (95.7) | | 33 (4.3) | <0.001 |
| Chest pain | 674 (98.1) | 13 (1.9) | 683 (88.4) | | 90 (11.6) | <0.001 |
| Swelling and pain in the lower limbs | 30 (4.4) | 657 (95.6) | 180 (23.3) | | 593 (76.7) | <0.001 |
| Fever | 655 (95.3) | 32 (4.7) | 721 (93.3) | | 52 (6.7) | 0.114 |
| Dizziness/Syncope | 679 (98.8) | 8 (1.2) | 674 (87.2) | | 99 (12.8) | <0.001 |
| Cough/Shortness of breath | 654 (95.2) | 33 (4.8) | 699 (90.4) | | 74 (9.6) | 0.001 |
| Palpitation | 682 (99.3) | 5 (0.7) | 742 (96.0) | | 31 (4.0) | <0.001 |
| Delirium/Disturbance of consciousness | 686 (99.9) | 1 (0.1) | 768 (99.4) | | 5 (0.6) | 0.278 |
| **Signs** |  |  |  | |  |  |
| Skin cold clammy | 680 (99.0) | 7 (1.0) | 746 (96.5) | | 27 (3.5) | 0.003 |
| Cyanosis of the lips | 686 (99.9) | 1 (0.1) | 754 (97.5) | | 19 (2.5) | <0.001 |
| Tachycardia | 651 (94.8) | 36 (5.2) | 664 (85.9) | | 109 (14.1) | <0.001 |
| Diminished respiration | 686 (99.9) | 1 (0.1) | 730 (94.4) | | 43 (5.6) | <0.001 |
| Pulmonary rales | 680 (99.0) | 7 (1.0) | 695 (89.9) | | 78 (10.1) | <0.001 |
| Accentuation/Splitting of P_2_ | 587 (85.4) | 100 (14.6) | 595 (77.0) | | 178 (23.0) | <0.001 |
| Distention of jugular vein/Hepatojugular reflex | 685 (99.7) | 2 (0.3) | 765 (99.0) | | 8 (1.0) | 0.161 |
| **ECG** |  |  |  | |  |  |
| Heart rate | 78.0(69.0, 89.0) | | 82.0 (72.0, 94.0) | | | <0.001 |
| S_Ⅰ_Q_Ⅲ_T_Ⅲ_ | 666 (96.9) | 21 (3.1) | 636 (82.3) | | 137 (17.7) | <0.001 |
| Nodal tachycardia | 633 (92.1) | 54 (7.9) | 670 (86.7) | | 103 (13.3) | 0.001 |
| Right ventricular hypertrophy | 687 (100.0) | 0 (0.0) | 760 (98.3) | | 13 (1.7) | 0.002 |
| Right axis deviation | 683 (99.4) | 4 (0.6) | 754 (97.5) | | 19 (2.5) | 0.008 |
| Left axis deviation | 632 (92.0) | 55 (8.0) | 597 (77.2) | | 176 (22.8) | <0.001 |
| S_1_S_2_S_3_ | 685 (99.7) | 2 (0.3) | 732 (94.7) | | 41 (5.3) | <0.001 |
| Low voltage | 670 (97.5) | 17 (2.5) | 738 (95.5) | | 35 (4.5) | 0.049 |
| Clockwise rotation of cardiac electric axis | 686 (99.9) | 1 (0.1) | 764 (98.8) | | 9 (1.2) | 0.042 |
| ST-segment elevation | 677 (98.5) | 10 (1.5) | 759 (98.2) | | 14 (1.8) | 0.744 |
| ST-segment depression | 654 (95.2) | 33 (4.8) | 682 (88.2) | | 91 (11.8) | <0.001 |
| T wave inversion(V_1_-V_3_/V_4_) | 653 (95.1) | 34 (4.9) | 598 (77.4) | | 175 (22.6) | <0.001 |
| ST-segment depression(Ⅱ/Ⅲ/aVF) | 673 (98.0) | 14 (2.0) | 702 (90.8) | | 71 (9.2) | <0.001 |
| Q/q wave(Ⅱ/aVF) | 670 (97.5) | 17 (2.5) | 699 (90.4) | | 74 (9.6) | <0.001 |
| T wave inversion(Ⅱ/aVF) | 681 (99.1) | 6 (0.9) | 717 (92.8) | | 56 (7.2) | <0.001 |
| Right bundle branch block | 662 (96.4) | 25 (3.6) | 723 (93.5) | | 50 (6.5) | 0.02 |
