# Supplementary material for: Development and validation of a prediction model to estimate risk of acute pulmonary embolism in deep vein thrombosis patients
Source: Sci Rep. 2022 Jan 13;12:649. doi: 10.1038/s41598-021-04657-y (PMC8758720; doi:10.1038/s41598-021-04657-y)
Supplement: Supplementary file 2 — Supplementary Table 2. [file 41598_2021_4657_MOESM2_ESM.docx]

Appendix Table 2 Predictiors with significant differences in training set and testing set

| **Characteristic**  n (%) or median (IQR) | **Train** | | **Test** | |
| --- | --- | --- | --- | --- |
|  | DVT  (n=517) | DVT+PE  (n=578) | DVT  (n=170) | DVT+PE  (n=195) |
| Age | 60 (48, 69) | 62 (51, 70) | 57 (48, 65) | 62 (51, 70) |
| **Pre-existing disease or condition** |  |  |  |  |
| Respiratory failure | 1 (0.2) | 23 (4.0) | 0 (0) | 8 (4.1) |
| Previous history of VTE | 55 (11) | 35 (6.1) | 21 (12) | 8 (4.1) |
| Malignant tumor | 62 (12) | 44 (7.6) | 15 (8.8) | 15 (7.7) |
| **Risk factors** |  |  |  |  |
| Infection | 19 (3.7) | 68 (12) | 8 (4.7) | 28 (14) |
| Superficial venous thrombosis | 11 (2.1) | 6 (1.0) | 8 (4.7) | 0 (0) |
| Long time of sitting(>6 hours) | 59 (11) | 23 (4.0) | 22 (13) | 6 (3.1) |
| **Symptoms** |  |  |  |  |
| Dyspnea | 21 (4.1) | 220 (38) | 7 (4.1) | 82 (42) |
| Hemoptysis | 3 (0.6) | 25 (4.3) | 0 (0) | 8 (4.1) |
| Chest pain | 11 (2.1) | 69 (12) | 2 (1.2) | 21 (11) |
| Swelling and pain in the lower limbs | 494 (96) | 441 (76) | 163 (96) | 152 (78) |
| Syncope | 6 (1.2) | 62 (11) | 2 (1.2) | 37 (19) |
| Cough | 30 (5.8) | 45 (7.8) | 3 (1.8) | 29 (15) |
| Palpitation | 4 (0.8) | 23 (4.0) | 1 (0.6) | 8 (4.1) |
| **Signs** |  |  |  |  |
| Skin cold clammy | 6 (1.2) | 21 (3.6) | 1 (0.6) | 6 (3.1) |
| Cyanosis of the lips | 1 (0.2) | 13 (2.2) | 0 (0) | 6 (3.1) |
| Tachycardia | 28 (5.4) | 87 (15) | 8 (4.7) | 22 (11) |
| Diminished respiration | 1 (0.2) | 30 (5.2) | 0 (0) | 13 (6.7) |
| Pulmonary rales | 4 (0.8) | 54 (9.3) | 3 (1.8) | 24 (12) |
| Accentuation/Splitting of P_2_ | 79 (15) | 133 (23) | 21 (12) | 45 (23) |
| **ECG** |  |  |  |  |
| Heart rate | 78 (69, 89) | 82 (73, 94) | 78 (71, 89) | 81 (70, 94) |
| S_Ⅰ_Q_Ⅲ_T_Ⅲ_ | 16 (3.1) | 93 (16) | 5 (2.9) | 44 (23) |
| Nodal tachycardia | 40 (7.7) | 77 (13) | 14 (8.2) | 26 (13) |
| Right ventricular hypertrophy | 0 (0) | 7 (1.2) | 0 (0) | 6 (3.1) |
| Right axis deviation | 2 (0.4) | 14 (2.4) | 2 (1.2) | 5 (2.6) |
| Left axis deviation | 39 (7.5) | 126 (22) | 16 (9.4) | 50 (26) |
| S_1_S_2_S_3_ | 1 (0.2) | 27 (4.7) | 1 (0.6) | 14 (7.2) |
| Low voltage | 13 (2.5) | 26 (4.5) | 4 (2.4) | 9 (4.6) |
| Clockwise rotation of cardiac electric axis | 1 (0.2) | 7 (1.2) | 0 (0) | 2 (1.0) |
| ST-segment depression | 27 (5.2) | 63 (11) | 6 (3.5) | 28 (14) |
| T wave inversion(V_1_-V_3_/V_4_) | 26 (5.0) | 131 (23) | 8 (4.7) | 44 (23) |
| ST-segment depression(Ⅱ/Ⅲ/aVF) | 13 (2.5) | 48 (8.3) | 1 (0.6) | 23 (12) |
| Q/q wave(Ⅱ/aVF) | 11 (2.1) | 50 (8.7) | 6 (3.5) | 24 (12) |
| T wave inversion(Ⅱ/aVF) | 6 (1.2) | 28 (4.8) | 0 (0) | 28 (14) |
| Right bundle branch block | 22 (4.3) | 33 (5.7) | 3 (1.8) | 17 (8.7) |
