# Supplementary material for: Development and validation of a prediction model to estimate risk of acute pulmonary embolism in deep vein thrombosis patients
Source: Sci Rep. 2022 Jan 13;12:649. doi: 10.1038/s41598-021-04657-y (PMC8758720; doi:10.1038/s41598-021-04657-y)
Supplement: Supplementary file 3 — Supplementary Table 3. [file 41598_2021_4657_MOESM3_ESM.docx]

Appendix Table 3 Multiple Logistic regression to construct a predictive model of acute pulmonary embolism

| **Characteristic** | **Coefficient** | **S.E.** | **OR** | **95% CI for OR** | | ***p-value*** |
| --- | --- | --- | --- | --- | --- | --- |
|  |  |  |  | **Lower** | **Upper** |  |
| **Pre-existing disease or condition** |  |  |  |  |  |  |
| Previous history of VTE | -0.88 | 0.30 | 0.41 | 0.23 | 0.73 | 0.00 |
| Respiratory failure | 1.90 | 1.09 | 6.66 | 1.15 | 127.99 | 0.08 |
| **Risk factors** |  |  |  |  |  |  |
| Infection | 0.88 | 0.33 | 2.41 | 1.27 | 4.68 | 0.01 |
| Superficial venous thrombosis | -1.47 | 0.72 | 0.23 | 0.05 | 0.84 | 0.04 |
| Long time of sitting (>6 hours) | -0.92 | 0.30 | 0.40 | 0.22 | 0.70 | 0.00 |
| **Symptoms** |  |  |  |  |  |  |
| Dyspnea | 2.41 | 0.28 | 11.11 | 6.51 | 19.91 | <0.00 |
| Hemoptysis | 1.96 | 0.72 | 7.10 | 1.94 | 34.93 | 0.01 |
| Syncope | 1.37 | 0.50 | 3.94 | 1.57 | 11.40 | 0.01 |
| Cough | -1.21 | 0.36 | 0.30 | 0.15 | 0.60 | 0.00 |
| **Signs** |  |  |  |  |  |  |
| Skin cold clammy | 0.77 | 0.56 | 2.17 | 0.75 | 6.86 | 0.16 |
| Tachycardia | 0.71 | 0.27 | 2.03 | 1.20 | 3.47 | 0.01 |
| Diminished respiration | 1.77 | 1.09 | 5.87 | 1.00 | 112.86 | 0.11 |
| Pulmonary rales | 1.06 | 0.61 | 2.90 | 0.96 | 11.12 | 0.08 |
| Accentuation/Splitting of P_2_ | 0.57 | 0.19 | 1.76 | 1.21 | 2.58 | 0.00 |
| **ECG** |  |  |  |  |  |  |
| S_Ⅰ_Q_Ⅲ_T_Ⅲ_ | 0.96 | 0.33 | 2.62 | 1.40 | 5.11 | 0.00 |
| Right axis deviation | 1.52 | 0.88 | 4.55 | 0.91 | 34.11 | 0.09 |
| Left axis deviation | 1.20 | 0.23 | 3.31 | 2.13 | 5.22 | 0.00 |
| S_1_S_2_S_3_ | 2.82 | 1.07 | 16.70 | 3.02 | 314.83 | 0.01 |
| T wave inversion(V_1_-V_3_/V_4_) | 0.58 | 0.27 | 1.79 | 1.05 | 3.09 | 0.03 |
| Q/q wave(Ⅱ/aVF) | 0.92 | 0.41 | 2.51 | 1.15 | 5.77 | 0.02 |
| Constant | -0.83 | 0.10 | 0.44 | 0.36 | 0.53 | 0.00 |
