# Supplementary material for: Development and validation of a prediction model to estimate risk of acute pulmonary embolism in deep vein thrombosis patients
Source: Sci Rep. 2022 Jan 13;12:649. doi: 10.1038/s41598-021-04657-y (PMC8758720; doi:10.1038/s41598-021-04657-y)
Supplement: Supplementary file 4 — Supplementary Information 1. [file 41598_2021_4657_MOESM4_ESM.pdf]

西安交通大学医学院第一附属医院

伦理委员会审查批件

受理号:KYLLSL-2018-105　　　　批件号：　2018 伦审科字第（144）号

No: XJTU1AF2018LSK-144

<table>
<tr><td>项目名称</td><td colspan="5">下肢深静脉血栓并发肺栓塞的发生机制及预后的预测研究</td></tr>
<tr><td>项目来源</td><td colspan="5">自选课题</td></tr>
<tr><td>负责单位</td><td colspan="5">西安交通大学第一附属医院</td></tr>
<tr><td>承担科室</td><td>周围血管科</td><td>项目负责人</td><td>田红燕</td><td>职称</td><td>主任医师</td></tr>
<tr><td>审查方式</td><td colspan="5">快速审查 □　　　会议审查　■</td></tr>
<tr><td>送审材料</td><td colspan="5">1、 伦理审查申请表
2、 患者知情同意书
3、 研究方案</td></tr>
<tr><td rowspan="3">审查结论</td><td>同意</td><td>作必要修正后同意</td><td>作必要修正后重审</td><td>不同意</td><td>终止或暂停</td></tr>
<tr><td>✓</td><td></td><td></td><td></td><td></td></tr>
<tr><td colspan="5">伦理会意见：
1 经审查，本项目修订后研究方案和知情同意书等设计合理，符合伦理原则，同意继续本研究。
2 该研究进行过程中，伦理委员会进行定期跟踪审查，审查频率：
3 个月□　　6 个月□　　12 个月■

主任委员：　　　　时间：　2018 年 10 月 19 日</td></tr>
<tr><td>注意</td><td colspan="5">1 对已批准的临床研究方案、知情同意书等材料的任何修改及主要研究者更换等。请及时通知本伦理委员会重新审查，获得批准后执行。
2 根据本伦理委员会的定期跟踪审查频率，请在审查日到期前一个月提交定期跟踪审查报告。
3. 发生严重不良事件及时报告。暂停/提前终止临床研究或项目结束，请提交相应的报告。
4. 本审查结果只涉及对伦理问题的审查结论，如相关研究要求办理相应手续，如到上级部门办理审批/备案手续，或按医院要求需要签署合同书/协议书的，请在项目开展前先行办理上述手续。</td></tr>
</table>

本伦理委员会严格遵循 ICH-GCP、GCP 和相关法规的要求进行构建、运作、实施各项操作程序。联系地址：西安市雁塔西路 277 号联系人：张彩霞，　　电话/传真：　85323473
